# Age and sex affect circadian patterns of cardiac autonomic function

**DOI:** 10.1038/s41598-025-18525-6

**Published:** 2025-09-29

**Authors:** Natalia Buitrago-Ricaurte, Alain Riveros-Rivera, Andre J. Riveros

**Affiliations:** 1 Escuela de Medicina y Ciencias de la Salud, Universidad del Rosario, Bogotá, Colombia; 2 Departamento de Ciencias Fisiológicas, Facultad de Medicina, Pontificia Universidad Javeriana, Bogotá, Colombia; 3 Departamento de Biología, Escuela de Ciencias e Ingeniería, Universidad del Rosario, Bogotá, Colombia; 4 Department of Neuroscience, College of Science, University of Arizona, Tucson, AZ 85721 USA

**Keywords:** Parasympathetic, Sympathetic, Circadian rhythm, Sex characteristics, Heart rate determination, Autonomic nervous system, Autonomic nervous system, Circadian mechanisms, Ageing

## Abstract

The cardiovascular system exhibits circadian rhythmicity, responding to central and peripheral clocks. Sexual dimorphism and aging independently influence circadian rhythms and cardiac electrical activity, which the autonomic nervous system regulates. However, the extent to which sex and aging affect autonomic dynamics remains unclear. This study investigates their role in circadian cardiac autonomic activity in healthy subjects. Data from 51 males and 51 females with 24-hour Holter recordings were analyzed. Circadian patterns of Heart Rate Variability parameters, indicators of autonomic function, were assessed using fast Fourier transformation with 5-minute Blackman-Harris windows on artifact-free recordings. Fractal, multifractal, visual, and information methods were applied to evaluate parasympathetic and sympathetic influences. We calculated the means and standard deviation for all numeric variables. We found temporal fluctuations across the time domain and frequency parameters. Six of the eight nonlinear HRV parameters significantly fluctuated. Among the 17 fluctuating parameters analyzed, nine circadian indicators (MESOR, amplitude, and acrophase) showed significant differences between sexes, with women exhibiting higher vagal oscillatory activity. Aging diminished fluctuations across all parameters and was associated with chronodisruption of cardiac autonomic markers. These findings highlight neurohumoral differences as key modulators of cardiac autonomic dynamics. The interaction effect of sex and age impacts the circadian rhythmicity of cardiac electric activity, as reflected by the HRV analysis. Our results have implications for personalized diagnostics, treatment timing, risk prediction, and biomarkers, highlighting the need for age-and sex-specific approaches in precision medicine and chronomedicine.

**Supplementary Information:**

The online version contains supplementary material available at 10.1038/s41598-025-18525-6.

## Introduction

The physiological processes of the cardiovascular system exhibit circadian rhythmicity in response to interactions between central and peripheral clocks. Central clocks operate through transcription-translation feedback loops that modulate the cortisol secretion, the activities of the autonomic nervous system and the renin-angiotensin-aldosterone axis^1^. Peripheral clocks occur in most cardiac cell lines, such as cardiomyocytes, endothelial cells, fibroblasts, and smooth muscle cells, and regulate transcription-translation loop expression of approximately 10 to 30% of genes in cardiomyocytes^2^. Together, the activity of these molecular clocks is ultimately evident at the systemic level, in the 24-hour rhythmic changes that occur in blood pressure, heart rate, cardiac contraction, and metabolic functions^3,4^. Importantly, such circadian rhythmicity ensures the maintenance of cardiovascular homeostasis, with deviations being associated with pathological processes^5^.

Within the proxies of cardiac electrical activity, auricular and ventricular depolarization exhibit a robust circadian pattern^6^. Studies in animal models suggest that the temporal expression of cardiac clock genes underlies these circadian oscillations, which in turn are related to the cyclic expression of ionic channel proteins^7^. For instance, the oscillatory expression of HCN4 mRNA mediated by CRY and PER (both involved in the determination of rhythmicity in physiological patterns) in the sinus node coincides with increases in heart rate^8^. This gene expression, in turn, is modulated by multiple neurohumoral factors and the influence of the central clock activity, leading to the rhythmic expression of cardiac ion channels throughout the day and resulting in variations in heart rate^6,9^. Hence, cardiac electrical activity during the circadian cycle is the product of the interaction of interoceptive and exteroceptive processes that regulate cardiac and central oscillators^10^.

The circadian oscillations in cardiac electrical activity are closely mirrored by Heart Rate Variability (HRV), a proxy derived from fluctuations in the time intervals between consecutive heartbeats^11^. As a reflection of autonomic activity dynamics, HRV enables inference of the predominance of the sympathetic or parasympathetic subsystems in diverse energetic contexts. For example, parasympathetic-related indices (RMSSD, pNN50, and HF) increase early in the morning and then decrease throughout the day^12^. In contrast, indices of total autonomic activity, such as LF/HF and SD2/SD1, increase during the day and decrease throughout the night^12^. Thus, HRV is a noninvasive valuable strategy for assessing autonomic balance in both healthy and diseased contexts.

Importantly, sexual dimorphism and age are independently associated with variations in circadian rhythms, thus becoming critical for a complete understanding of distinct patterns in cardiovascular function. On the one hand, sex effects on rhythmicity may contribute to variations in cardiac electrical activity and susceptibility to specific pathological conditions^13^. For example, entrainment in females and males differs in terms of melatonin, temperature, and heart rate^14^, likely due to particularities of the sleep-wake cycle between sexes^15^. Additionally, circadian-related genes are modulated by estrogen and testosterone, resulting in differential protein expression in females and males^16,17^. Thus, sexual circadian dimorphism is an important issue and recognizing these differences may help tailor medical treatments and promote personalized healthcare strategies^18^.

On the other hand, aging significantly affects the molecular, cellular, and functional circadian mechanisms of the heart. Functionality decreases with age, including lower contractility, ventricular relaxation capacity, and maximum heart rate^19^. In older subjects, heart rate exhibits reduced variability, increased regularity, decreased complexity, and diminished vagal influence during 24-hour cycle fluctuations^20^. Senescence alters autonomic function through mechanisms related to reduced intracellular calcium transport, decreased adrenergic responsiveness, and differential expression of clock-related genes in the heart^21^. These senescence-related changes underscore the intricate interplay between circadian biology and cardiac function, highlighting the importance of understanding these mechanisms to address the age-associated cardiovascular risk phenotype.

As described above, age and sex impact the dynamics of cardiac electrical activity, which is regulated primarily by the autonomic nervous system. These dynamics, led by changes in sympathetic and parasympathetic predominance, are further influenced by circadian oscillations. However, the interaction between these factors, namely, whether and how the variation in autonomic dynamics is influenced by sex and aging, remains unclear. This is critical as these interactions significantly impact the clinical presentation, development, and prognosis. Hence, as a first step, we aimed to test whether age and sex are associated with variations in circadian cardiac autonomic activity in healthy subjects. We rely on time- and frequency-domain parameters to evaluate autonomic balance, and nonlinear indices to characterize system complexity, adaptability, and self-regulatory capacity, offering complementary insights into autonomic and circadian regulation.

## Methods

### Subjects and data collection

We conducted a retrospective study that relied on data from 102 individuals (51 males, 51 females) obtained from the Telemetric and Holter ECG Warehouse database from the University of Rochester Medical Center^22^. Following established guidelines, we estimated the sample size via G*Power software, with a statistical power of 0.95 and an alpha error probability of 0.05^23^. The database originally included subjects complying with the following inclusion criteria (see also Ref Thew Database):

1. No overt cardiovascular disease or history of cardiovascular disorders (including stroke, TIA, peripherical vascular disease).
2. No history of high blood pressure (> 150/90).
3. No medication.
4. No chronic illness (e.g., diabetes, asthma, chronic obstructive pulmonary disease, etc.).
5. The subject was not enrolled if s/he was evaluated by a physician for the cardiovascular-related syndrome (chest pain, palpitation, syncope) but was otherwise diagnosed as being healthy.
6. No abnormal physical examination.
7. Sinus rhythm in 12-lead ECG without any suspicious abnormalities (e.g., signs of ventricular hypertrophy, inverted T-wave, intraventricular conduction disturbances).
8. Normal echo and normal ECG exercise testing in presence of suspicious ECG changes.
9. No pregnancy.

The recordings were originally acquired using the SpaceLab-Burdick digital Holter recorder (SpaceLab-Burdick, Inc., Deerfield, WI). Before starting the ambulatory recording under routine daily living conditions, the researchers allowed the subjects a supine resting period of 20 min. Then, 24-hour ambulatory monitoring of electrocardiogram activity was conducted by using three leads with a pseudoorthogonal configuration (X, Y and Z)^24,25^. The sampling frequency was 200 Hz, and the amplitude resolution was 10 µV. The recordings were visually supervised and purified manually for artifacts by a specialized technician using Vision Premier (SpaceLab-Burdick, Inc., Deerfield, WI).

**Fig. 1:**
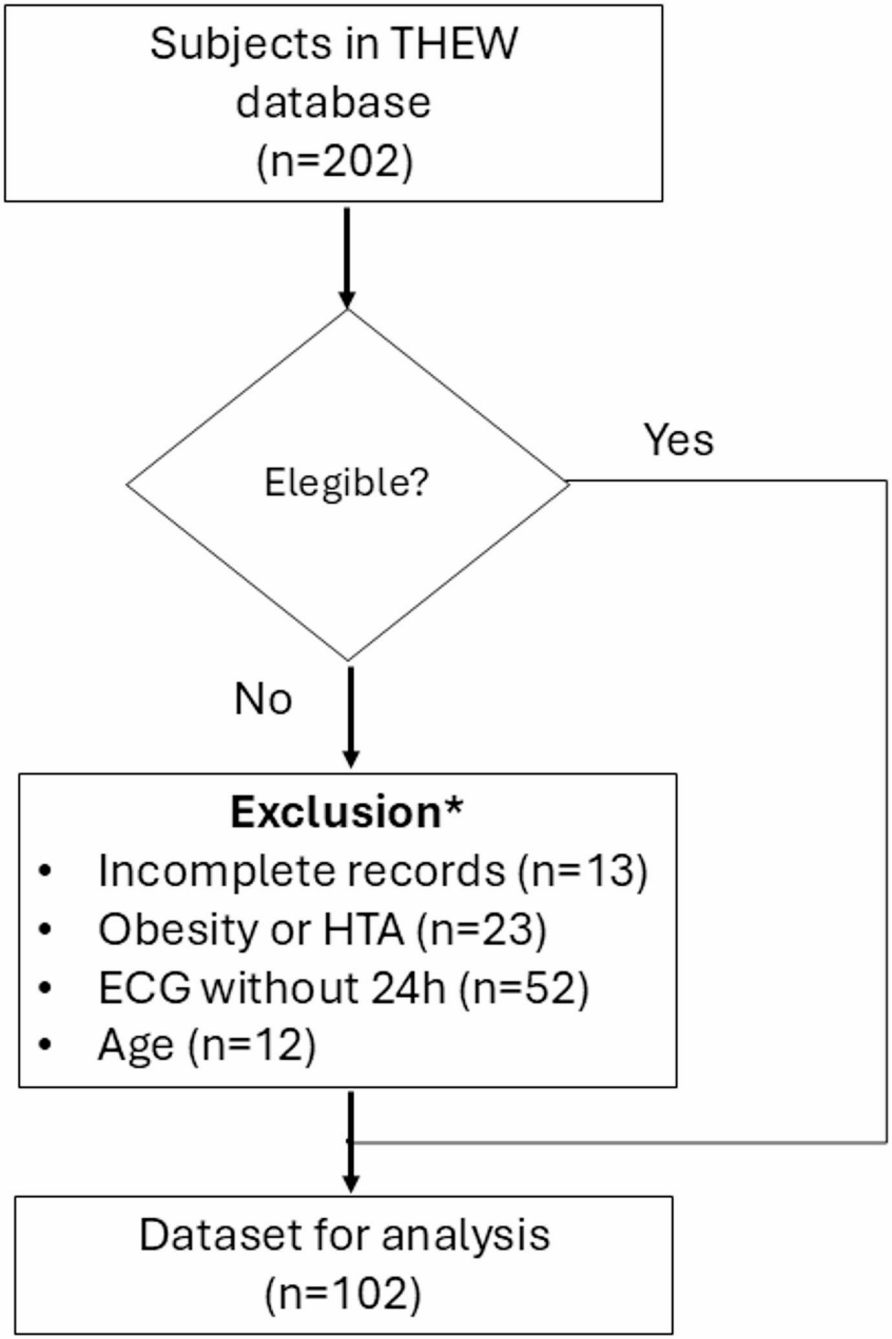
Flowchart diagram for the selection of subjects.

Once we acquired this database, we selected subjects on the basis of the following additional criteria: data recordings of at least 24 h and data quality according to the noise detection score determined by the software *Kubios* (see below; v.4.2.0, Kubios, OY). We subsequently, conducted a retrospective observational study including 102 subjects (Fig. 1). We determined the analysis groups: “males”, “females”; we further classified individuals as “young” or “old” on the basis of the mean menopausal age of our population ≈ 50 years. We also included the following variables for further analysis: Body Mass Index (BMI), smoking status, and systolic and diastolic blood pressure.

### Analysis of heart rate variability

#### Preprocessing

We used the preprocessing tools available from Kubios HRV Scientific. Specifically, according to HRV standards, we identified RR intervals and selected free-artifact windows for five minutes *per* hour^26^ and then used tools for noise detection, beat correction, and sample selection; thus, we aimed to identify arrhythmias and correct misidentifications and artifacts^27^. We excluded 15 recordings due to strong threshold beat correction (excluding > 5% of total beats). Three analyzed recordings required beat correction with heartbeats corrected within 1% and 3%. We first assessed the stationarity of the RR interval signals by identifying and removing artifacts via noise detection and threshold-based beat correction algorithms at Kubios HRV Scientific. We subsequently, applied the smoothness prior detrending method to eliminate slow nonstationary trends. Only segments classified as stationary on the basis of this processing and guided by the respiratory rate estimation (RESP) feature were retained for further analysis.

#### Analysis in the time domain

We identified each QRS complex and selected the RR intervals between two normal complexes (hereafter NN intervals) based on a cubic spline interpolation filter with a maximal correction factor of 20% using Kubios HRV Scientific and based on the Task Force standards^28^. We obtained four indices in the time-domain: (i) standard deviation of all NN intervals (SDNN), (ii) Standard deviation of the average NN intervals (SDANN), (iii) Root Mean Square of the difference between successive NN intervals (RMSS), and (iv) The proportion of adjacent normal NN intervals differing by more than 50 ms (pNN50).

#### Analysis in the frequency domain

We conducted a fast Fourier transformation using 5 min Blackman-Harris windows for the preprocessed artifact-free recordings (number of segments per hour: 12, points per frequency domain: 300 points/Hz, interpolation rate: 4 Hz). We obtained the power densities by integrating the power spectral density in each frequency band: high frequency (HF: 0.15–0.5 Hz), low frequency (LF: 0.04–0.15 Hz), and very low frequency (VLF: 0.0033–0.04 Hz)^26^. We also calculated the LF/HF index and expressed it in a normalized power spectrum.

#### Analysis of nonlinear parameters

To explore the complex dynamics of autonomic regulation, we analyzed fractal and multifractal (Detrended Fluctuation Analysis-Alf1, Alf2,CorrDim), geometric (Poincare plot-SD1, SD2), entropy-based (Approximate Entropy, Sample Entropy, Shannon Entropy) and recurrence measures (REC-DET)^29,30^.

#### Autonomic indices

We analyzed three parameters derived from HRV metrics that reflect the predominance of the parasympathetic and sympathetic branches of the autonomic nervous system, following the methodology outlined in the white paper provided by Kubios HRV software^31^. Each parameter was normalized via z-scores relative to a reference population and reported accordingly^31^. Significant autonomic activity was considered when values exceeded ± 1 standard deviation from the normative range. The Parasympathetic Nervous System Index (PNSi) is computed based on the mean RR interval, RMSSD, and Poincaré SD1 all of which are markers of parasympathetic activity. The Sympathetic Nervous System Index (SNSi) integrates the mean heart rate (HR), the Baevsky’s Stress Index^32^, and the Poincaré plot index SD2 which are associated with sympathetic dominance. Finally, the Stress Index (STi) is calculated as the square root of Baevsky’s Stress Index, providing a nonlinear measure of cardiovascular stress.

### Analysis of circadian rhythmicity

We analyzed whether Heart Rate Variability (HRV) parameters exhibited a circadian pattern over the 24-hour recording period. We first evaluated the uniformity of the temporal distribution using cross-validation between repeated measures ANOVA and the FFT-NNLS method^33^. Circadian oscillation and rhythmicity were assessed by fitting a cosinor model to the hourly means of the HRV variables. The model is defined as: Y(t) = *M* + *A*⋅cos(2π/*T* + ϕ), where *M* is the mesor (rhythm-adjusted mean), *A* is the amplitude, *T* is the assumed period (24 h), and ϕ is the acrophase. The circadian parameters (MESOR, amplitude, and acrophase) were estimated via the **‘card’** package in R^34^. When rhythmicity was detected and circadian parameters were calculated, we further explored group differences (e.g., by sex or age group) in these parameters. If extant, we further evaluated potential differences between groups (females, males, young, and old) in these variables.

### Statistical analyses

We conducted descriptive statistical analyses to characterize the sample. Continuous variables are reported as the means and standard deviations, whereas categorical variables (e.g., race and smoking status) are presented as absolute frequencies. Group comparisons (Females vs. Males, Young vs. Old) were performed using Chi-Squared tests for categorical variables and Student’s t-tests or Mann-Whitney U tests for continuous variables, depending on the normality of distributions assessed via the Kolmogorov-Smirnov test.

We analyzed 19 HRV variables, including time-domain (*n* = 3), frequency-domain (*n* = 4), nonlinear dynamics (*n* = 9), and autonomic indices (*n* = 3). Circadian rhythmicity was first assessed through a uniformity test based on cross-validation between repeated measures ANOVA and the FFT-NNLS method^35^. Upon confirmation of rhythmicity, Cosinor analysis was used to estimate key circadian parameters: MESOR (mean level), amplitude, and acrophase (ϕ)^25,26^.

Group comparisons of Cosinor parameters were performed using appropriate parametric or nonparametric tests. Post hoc corrections for multiple comparisons were applied using the Cliff method. Furthermore, we performed multivariable logistic regression to estimate odds ratios and 95% confidence intervals, assessing the influence of age, sex, and BMI on the presence of rhythmicity. To evaluate the interaction effects between age and sex on circadian parameters, we used a three-way ANOVA. The sample size determination, as reported in the subjects section, was calculated and guided by previous HRV circadian studies, ensuring sufficient statistical power (> 80%) to detect meaningful differences in MESOR and acrophase between groups (Supplementary Figure S3).

All the statistical analyses were conducted using the card package in R Statistical Software (v4.1.2, R Core Team 2021) and JMP^®^ Pro software (v18, SAS Institute Inc., Cary, NC, USA). A two-tailed p-value < 0.05 was considered statistically significant.

### Ethical considerations and approval

The participants provided informed consent following the specified criteria of the Telemetric and Holter ECG Warehouse database at the University of Rochester Medical Center^22^. All data contained in the THEW were intended to be deidentified for the Health Insurance Portability and Accountability Act of 1996 (“HIPAA”) and the regulations promulgated thereunder. All methods were performed according to the relevant guidelines and regulations.

This study received approval from the Ethical Committee of the Instituto del Corazón de Bucaramanga, Sede Bogotá, Colombia, under the Helsinki Declaration (protocol number 06, November 8, 2023).

## Results

### Subjects

We analyzed 51 males and 51 females without reported comorbidities and with continuous use of medications. We only detected differences in the median BMI (males: median ± IQR: 25.5 ± 2.8, females: 24.1 ± 3.0; see Table 1 for results across all variables and Supplementary Figure S1 for histograms and descriptive statistics of age).

**Table 1:** Baseline characteristics between females and males. *Mann Whitney test: U = 1720, *p* = 0.005, *r* = 0.32. IQR = Inter quartile Range.

	Females (*n* = 51)	Males (*n* = 51)	*p*
Age (median ± IQR)	49.5 ± 13.0	45.7 ± 13.9	0.102
White ethnicity (n-%)	48-94.1%	48-94.1%	0.721
BMI (median ± IQR)	24.1 ± 3.0	25.5 ± 2.8	0.005*
Systolic blood pressure (median ± IQR)	121.4 ± 14.4	122.9 ± 11.6	0.416
Diastolic blood pressure (median ± IQR)	77.2 ± 6.9	77.9 ± 7.2	0.99
Smokers (n-%)	18-35.2%	21-41.1%	0.54

### Heart rate variability

#### Assessment of oscillation and detection of rhythmicity

We found that in males, SamPEN and ShannE exhibited a uniform distribution of data (one-way repeated measures ANOVA: SamPEN: F_23,101_ = 1.76, *p* = 0.185; ShannE: F_23,101_ = 0.02, *p* = 0.886). Among females, only ShannE exhibited a uniform distribution (one-way repeated measures ANOVA: F_23,101_ = 0.079, *p* = 0.779). We further confirmed the oscillatory pattern from visual inspection of the spectrograms. The fulfillment of the criterion of uniformity allowed us to proceed with the cosinor analysis (see next).

**Fig. 2:**
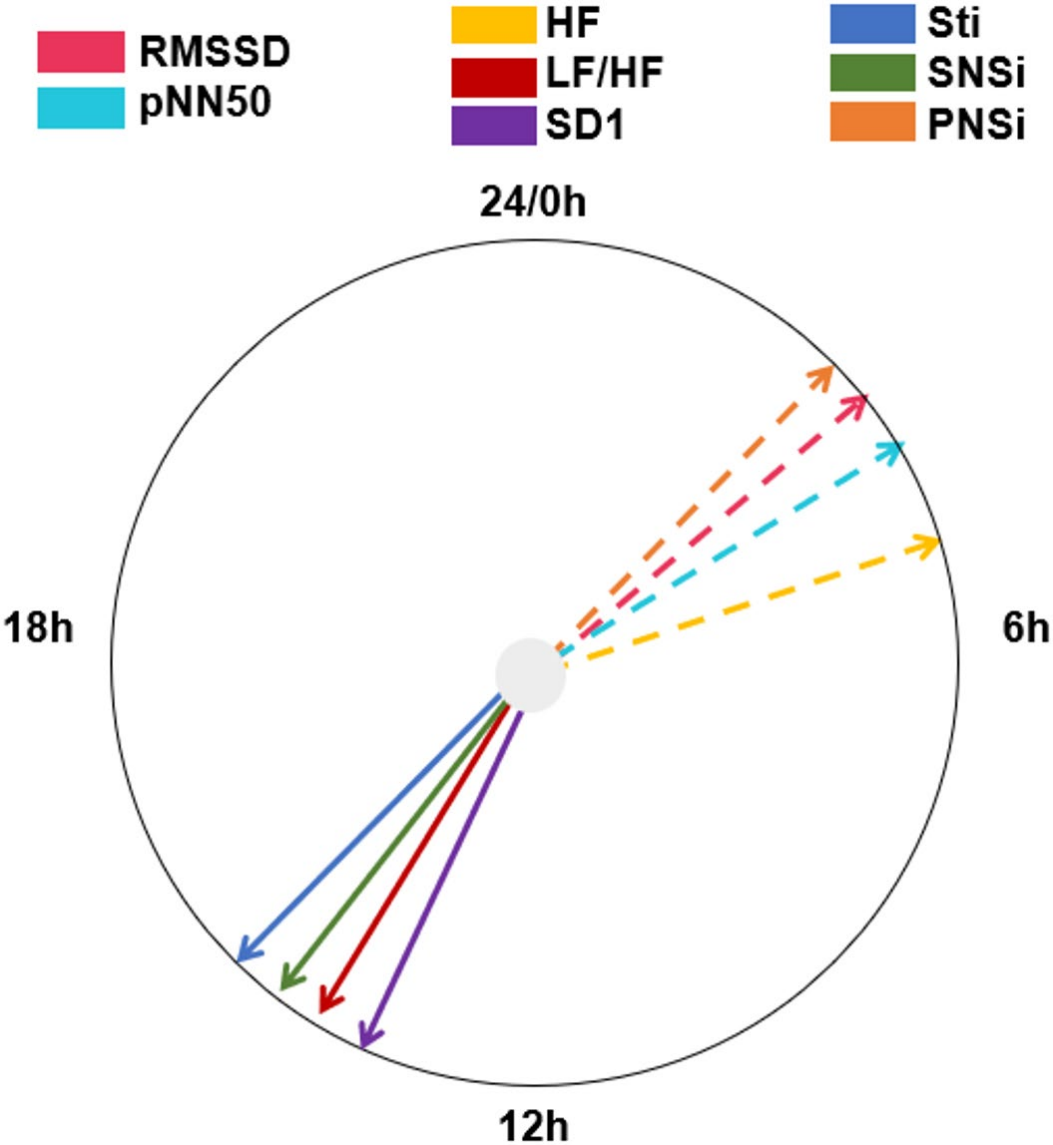
Distribution of acrophases for indicators of sympathetic (solid line) and parasympathetic predominance (dashed line).

We found that the time-domain, frequency-domain, and nonlinear HRV parameters exhibited circadian rhythmicity (see Supplementary Table S1). Additionally, the acrophase, indicating the peak time of the cyclic expression of a variable, was different for proxies associated with sympathetic or parasympathetic activity (Fig. 2).

#### Variation in circadian parameters between sexes

**Fig. 3:**
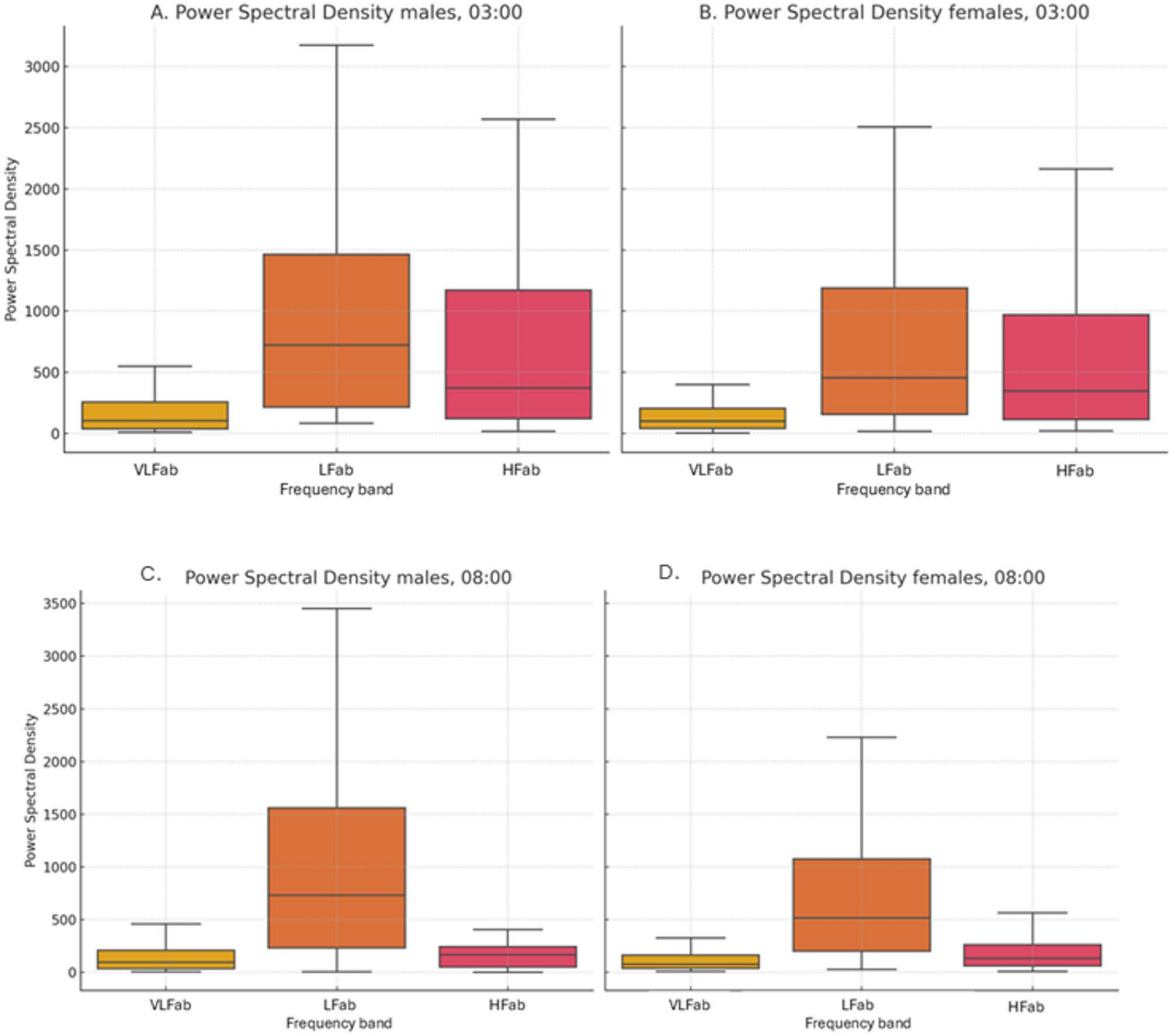
Power spectral density (PSD) boxplots comparing males and females at key circadian time points. (**A**) PSD in males at 03:00; (**B**) PSD in females at 03:00; (**C**) PSD in males at 08:00; (**D**) PSD in females at 08:00. Each boxplot represents the distribution of spectral power across subjects, highlighting sex-related differences in autonomic modulation at these specific circadian phases. PSD was computed from 5-minute RR interval segments using Welch’s method.

We found that females exhibited a lower MESOR in the SDNN, a parameter describing long-term autonomic activity (SDNN MESOR (mean ± SD): Females:32.89 ± 12.6ms; Males:39.8 ± 16ms; F_1,101_=4.83, *p* = 0.03), lower MESOR in VLF, (VLF MESOR (mean ± SD): Females:125 ± 72.9 ms^2^, Males:174.3 ± 99.3ms; F_1,101_=5.63, *p* = 0.01) and in three out of four related sympathetic activity indicators (LF MESOR (mean ± SD): Females: 732.5 ± 582.9ms; Males:1112 ± 790.7ms; F_1,101_=5.04, *p* = 0.02; LF/HF MESOR (mean ± SD): Females: 3.98 ± 2.1, Males:5.02 ± 2.59; F_1,101_=0.53, *p* = 0.02, SD2 MESOR (mean ± SD): Females: 40.99 ± 15.10ms; Males: 49.49 ± 17.9ms; F_1,101_=2.33, *p* = 0.02). Females presented a greater mean MESOR for HFn (Normalized High-Frequency Power), a parameter related to parasympathetic activity MESOR: Females:31.3 ± 10.7%; Males:27.2 ± 12.1%; F_1,101_=0.15, *p* = 0.01). Females presented a greater mean MESOR for AmpEn (MESOR (mean ± SD): Females: 1.11 ± 0.08; Males: 1.06 ± 0.0009; F_1,101_=14.9, *p* = 0.0002), a parameter indicating irregularity and complexity. Finally, males exhibited an earlier acrophase for the stress index than females (mean ± SD: Males: 16:43 ± 4 h; Females: 18:42 ± 5 h; F_1,101_=4.38, *p* = 0.03; Table 2; Fig. 3).

**Table 2:** Comparisons of MESOR, amplitude and acrophase between females and males for parameters of HRV in the time and frequency domains, the nonlinear parameters and the autonomic indices. All values are presented as the median [IQR].

Analysis	Variable	MESOR		Amplitude		Acrophase	
		Males	Females	Males	Females	Males	Females
Time domain	SDNN* (ms)	39.8 [16.1]^a^	32.9 [12.6]^a^	9.5 [8.9]	8.1 [6.1]	00:18 [6]	23:03 [7]
	RMSSD (ms)	34.7 [22.9]	28.8 [14.8]	13.1 [14.3]	11.5 [11.5]	02:24 [6]	23:36 [7]
	pNN50 (%)	11.7 [11.4]	8.2 [8.8]	7.9 [8.3]	6.7 [6.5]	02:12 [6]	00:18 [6]
Frequency domain	HFn (%)	27.2 [12.1]^b^	31.3 [10.7]^b^	9.7 [5.2]	11.3 [6.4]	02:50 [6]	00:12 [7]
	LFn (%)	72.7 [12.1]^a^	68.6 [10.7]^a^	9.7 [5.2]	11.3 [6.4]	18:18 [3]	19:17 [5]
	VLF (ms^2^)	174.3 [99.3]^a^	125 [72.9]^a^	89.3 [73.5]	67.7 [58.3]	00:45 [5]	23:36 [6]
	LF/HF	5.1 [2.5]^a^	3.9 [2.1]^a^	2.1 [1.5]	19 [1.1]	18:13 [3]	18:36 [5]
Nonlinear	SD1 (ms)	24.6 [16.2]	20.4 [10.5]	9.2 [10.1]	8.1 [8.1]	01:24 [6]	23:36 [7]
	SD2 (ms)	49.4 [17.9]^a^	40.9 [15.1]^a^	10.3 [8.7]	8.5 [5.8]	23:25 [6]	22:24 [7]
	SD2/SD1	2.5 [0.6]	2.4 [0.5]	0.4 [0.2]	0.4 [0.2]	19:28 [4]	18:58 [5]
	AmpEN	1.06 [0.009] ^c^	1.1 [0.08] ^c^	0.06 [0.03]	0.05 [0.02]	16:16 [6]	17:49 [6]
	Alf1	1.2 [0.2]	1.2 [0.2]	0.16 [0.09]	0.18 [0.1]	18:01 [3]	18:22 [4]
	Alf2	0.45 [0.1]^b^	0.49 [0.07]^b^	0.07 [0.03]	0.08 [0.035]	18:15 [3]	18:04 [5]
	CorrDim	1.6 [1.0]^a^	1.2 [0.8]^a^	0.5 [0.4]	0.6 [0.4]	22:55 [7]	23:43 [6]
	REC (%)	34.7 [6.5]	34.8 [7.2]	4.4 [2.3]	5.0 [3.8]	19:12 [5]	18:18 [5]
	DET (%)	98.1 [0.6]	97.9 [0.6]	0.6 [0.3]	0.6 [0.3]	18:18 [4]	17:35 [5]
Autonomic indices	SNS	1.18 [1.39]	1.61 [1.31]	1.09 [0.48]	1.15 [0.59]	17:55 [3]	18:38 [4]
	PNS	-0.59 [1.1]	-0.9 [0.7]	0.93 [0.64]	0.85 [0.58]	03:36 [4]	02:46 [5]
	Stress index (STi)	-3.78 [0.7]	-3.56 [1.1]	2.71 [1.7]	3.1 [1.7]	16:43 [4]^a^	18:42 [5]^a^

Statistically significant differences are indicated by superscripts a = *p* < 0.05 b = *p* < 0.01, c = *p* < 0.001. All values within the acrophase are in hours: minutes (hh: mm) and are not included to improve visualization. Units are presented in the ‘variable’ column. HFn: normalized High-Frequency Power-LFn: normalized Low-Frequency Power.

*Following the suggestion of monfredi et al.^36^, correction of this parameter by HR is included; the corrected values are: (SDNN MESOR (mdn ± IQR): females: 0.036 ± 0.024ms, males: 0.049 ± 0.047 ms), (SDNN amplitude (mdn ± IQR): females: 0.008 ± 0.01ms, males: 0.009 ± 0.01 ms) since comparisons did not affect the corrected values are not further discussed hereafter.

#### Variation in circadian parameters between age groups

We found that individuals in the group “Old’ exhibited lower overall and long-term variability, which occurred earlier in the day (SDNN MESOR (mean ± SD): Young: 40.8 ± 14.3ms, Old: 29.7 ± 13ms; F_1,101_=14.57, p = 0.0002; (Monfredi et al correction: Young: 0.058 ± 0.032ms; Old: 0.038 ± 0.02ms p = < 0.001), SDNN Amp (mean ± SD): Young: 10.3 ± 8.5ms, Old: 6.44 ± 5.5ms; F_1,101_=7.77, p = 0.006; (Monfredi et al correction: Young: 0.015 ± 0.01ms; Old: 0.008 ± 0.008ms p = < 0.005), SDNN Φ (mean ± SD): Young: 02.27 h ± 4 h, Old: 20:57 h ± 8 h; F_1,101_=12.9, p = 0.0005). Individuals in the group “Old” also exhibited lower overall autonomic activity, occurring earlier (LFab MESOR (mean ± SD): Young: 1194 ± 753ms², Old: 517 ± 408ms²; F_1,101_=28.75, p < 0.0001; LFab Amp (mean ± SD): Young: 452 ± 445ms², Old: 227 ± 753ms²; F_1,101_=12.84, p = 0.0006; SD2 MESOR (mean ± SD): Young: 51.6 ± 16.3ms, Old: 35.7 ± 13.2ms F_1,101_=27.21, p < 0.001; SD2 Amp (mean ± SD): Young: 11.1 ± 8ms, Old: 7.05 ± 5.8ms; F_1,101_=10.9, p = 0.002; SD2 Φ (mean ± SD): Young: 00:33 ± 5 h, Old: 20.46 ± 7 h; F_1,101_=8.24, p = 0.005).

Interestingly, we observed differences between age groups in the indicators of circadian rhythm (MESOR, amplitude and acrophase) across HRV parameters associated with sympathetic and parasympathetic related parameters. The MESOR and amplitude were lower, and the acrophase occurred earlier in older subjects across parameters associated with parasympathetic predominance (Table 3). In contrast, parameters associated with sympathetic predominance were greater and occurred earlier in subjects within the group “Old” (Table 4). The indices reflecting the sympathovagal balance occurred later in individuals in the group “Old” with subtle differences in the overall balance(LF/HF Φ (mean ± SD): Young: 17:26 ± 3 h, Old: 19:17 h ± 5 h; F_1,101_= 6.76, *p* = 0.01; SD2/SD1 Amp (mean ± SD): Young: 0.53 ± 0.25ms, Old: 0.39 ± 0.26ms; F_1,101_= 10.91, *p* = 0.01; SD2/SD1 Φ (mean ± SD): Young: 18:31 ± 3 h, Old: 20:38 h ± 5 h; F_1,101_=4.87, *p* = 0.02).

**Table 3:** Comparisons of indicators associated with parasympathetic predominance between age groups.

	RMSSD		pNN50		HFab		SD1		PNS	
	Young	Old	Young	Old	Young	Old	Young	Old	Young	Old
MESOR	ND	ND	13.1% ± 11.2^b^	5.2% ± 6.3^b^	628 ms^2^ ± 704^a^	306 ms^2^ ± 449^a^	ND	ND	ND	ND
Amplitude	14.6 ms ± 15^a^	8.7 ms ± 8^a^	9.8% ± 8.3^a^	3.6% ± 3.6^a^	547ms^2^ ± 894^a^	210 ms^2^ ± 399^a^	10.3 ms ± 10.6^a^	6.1 ms ± 5.7^a^	1.0 ms ± 0.7^b^	0.7 ms ± 0.3^b^
Φ	3:10 ± 3^b^	21:47 h ± 8^b^	3:12 h ± 4^a^	21:07 h h ± 8^a^	03:06 h ± 3^a^	23:24 h ± 7^a^	03:49 h ± 3^b^	21:1 h ± 8^b^	ND	ND

All values are presented as the mean ± sd. Statistically significant differences are indicated by superscripts a = *p* < 0.05, b = *p* < 0.001. ND = No significant differences. HFab: absolute High-Frequency power.

**Fig. 4:**
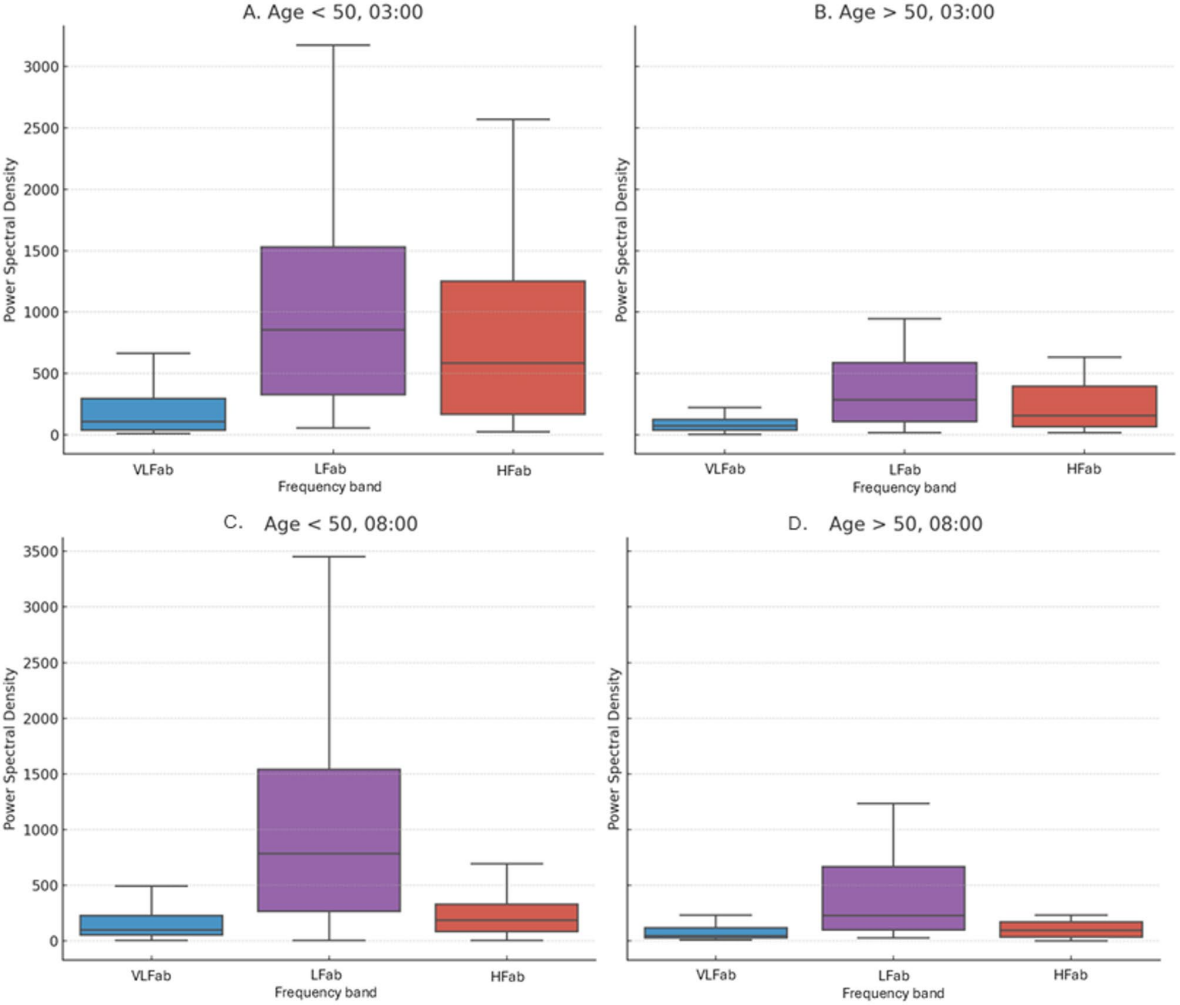
Power spectral density (PSD) boxplots comparing ‘Young’ and ‘Old’ groups at key circadian time points. (**A**) PSD in the Young group at 03:00; (**B**) PSD in the Old group at 03:00; (**C**) PSD in the Young group at 08:00; (**D**) PSD in the Old group at 08:00.

Each boxplot shows the distribution of spectral power across subjects within each age group, illustrating differences in autonomic modulation at distinct circadian phases. PSD was computed from 5-minute RR interval segments using Welch’s method.

**Table 4:** Comparisons of indicators associated with sympathetic related parameters between age groups. All values are presented as the mean ± sd.

	*SNS*		*STi*		*LFab*		*SD2*	
	Young	Old	Young	Old	Young	Old	Young	Old
MESOR	ND	ND	13.1 ± 11.2^b^	5.2 ± 6.3^b^	1194 ± 753^c^	517 ± 408 ^c^	51.6 ± 16.3^b^	35.7 ± 13.2^b^
Amplitude	14.6 ± 15^a^	8.7 ± 8^a^	9.8 ± 8.3^b^	3.6 ± 3.6^b^	452 ± 445^c^	227 ± 753^c^	11.1 ± 8^b^	7.05 ± 5.8^b^
Φ	3:10 h ± 3^b^	21:47 h ± 8^b^	3:12 h ± 4^a^	21:07 h ± 8^a^	ND	ND	00:33 h ± 5^b^	20:46 h ± 7^b^

Statistically significant differences are indicated by superscripts a = *p* < 0.05, b = *p* < 0.001. ND = No significant differences.

LFab: absolute low-frequency Power.

Finally, we found that younger individuals presented greater complexity with lower recurrence and regularity, (CorrDimD2 MESOR (mean ± SD): Young: 1.8 ± 0.9, Old: 0.8 ± 0.7; F_1,101_=31.09, *p* < 0.001; CorrDimD2 Amp (mean ± SD): Young: 0.7 ± 0.4, Old: 0.4 ± 0.3; F_1,101_= 13.06, *p* = 0.0005; REC MESOR (mean ± SD): Young: 32.7 ± 3.8, Old: 37.8 ± 8.9; F_1,101_=15.65, *p* = 0.0001; REC Amp (mean ± SD): Young: 3.9 ± 2.2, Old: 5.8 ± 4.0; F_1,101_=6.24, *p* = 0.01).

#### Combined effects of age and sex on circadian parameters

Finally, we found significant variation in two parameters of the HRV across sex and age groups. Specifically, we found that older woman presented lower and later sympathetic HRV related parameters than other groups did (SNS Φ (mean ± SD): Older female: 21:43 h ± 4 h; F_1,101_=10.09, *p* = 0.001; STI Φ (mean ± SD): Older female: 13.45 h ± 6 h; F_1,101_=5.74 *p* = 0.01).

## Discussion

Age and sex are factors that impact the dynamics of cardiac electrical activity, which are regulated primarily by the autonomic nervous system (ANS). These dynamics, led by the changes in sympathetic and parasympathetic predominance, are further influenced by circadian oscillations. Nevertheless, the interaction between these factors, namely, whether and how the variation in autonomic dynamics is influenced by sex and aging, remains unclear. This is critical as these interactions significantly impact the clinical presentation, development, and prognosis. Hence, as a first step, we aimed to test whether age and sex are associated with variations in circadian cardiac autonomic activity in healthy subjects. We found that aging was associated with lower circadian fluctuations, an advanced phase of autonomic oscillations, lower complexity and adaptability. Also, women exhibited a distinct pattern of sympathovagal balance, which further initiated later in the day. We argue that these findings can be explained by three interconnected factors: (i) senescence of tissue and cardiac oscillators, (ii) ANS-interoception, and (iii) hormonal effects on heart electrical activity.

First, the senescence of the cardiac tissue and circadian oscillators may explain the lower fluctuation and chronodisruption of the autonomic oscillations. In humans and animal models, senescent subjects exhibit a decline in cardiac functions (e.g., lower heart rate, contractility and cardiac output);^37–39^ and deterioration of the regulators of circadian patterns (e.g., less baroreceptor output, less afferent and efferent neuronal conduction, altered central integration and sinoatrial node responsiveness)^12,40^. In our population, senescence was associated with reduced autonomic outflow (Tables 3 and 4; Fig. 4), diminished long-term variability, and an overall decrease in signal complexity accompanied by increased recurrence of cardiac dynamics. Individuals in the “Old” group exhibited lower complexity, greater regularity, and recurrence, a pattern likely derived from lower overall and long-term HRV, and less vagal activity (Table 3; Fig. 4). Notably, aging was associated with a change in the circadian pattern of sympathetic related parameters, from an advanced phenotype in older individuals (Table 4). Clearly, the retrospective observational nature of our study does not allow mechanistic explanations for the observed patterns. Yet, our results align with the previous findings that autonomic aging is underlined by oxidative stress^41^, adrenergic desensitization^42^, and heart remodeling^43^, which induce an inflammatory and disbalanced sympathetic phenotype^42,44^.

Second, aging exerts an effect on the electrical circadian complexity dynamics of the heart. Disruption of the autonomic balance and a senescent circadian system impact electrical interdependence and redundancy^45^. For example, cardiovascular disease is characterized by decomplexification, increased regularity, and reduced variability of the electrical pattern^46^. This finding is consistent with our observation that young subjects presented greater complexity, less regularity, and recurrence (see results of HRV nonlinear parameters). Nonlinear HRV parameters, including entropy-based and recurrence measures, provide valuable insights into the regularity, temporal dynamics, and flexibility of cardiac autonomic signals. Moreover, senescence may alter autonomic function and the expression of clock-related genes in the heart, disrupting both interoceptive and exteroceptive pathways. These age-related changes may increase the threshold for physiological responsiveness, thereby impairing synchronization and further diminishing the regularity, temporal adaptability, and complexity of autonomic cardiac control. For example, in the heart, the expression of 148 genes with circadian patterns decreases during aging^47^. In the central nervous system, autonomic-related regions such as the amygdala, the nucleus accumbens, and the hypothalamus present blunted rhythmic activity with age^17^. Importantly, aging affects repolarization, similar to the effect caused by sex, and has a pronounced effect on both autonomic and local cardiac activity, leading to disruptions in the local and central circadian systems^48^.

Third, hormonal and genetic factors define distinct expression profiles between sexes and may further influence autonomic function and cardiac oscillators. On the one hand, estrogens act on ERα and ERβ in the neuronal bodies and axons of autonomic nuclei throughout the nervous system^49^. For example, 17β -estradiol modulates synaptic sympathetic transmission^50,51^, regulating the activity of central and peripheral controllers of autonomic cardiac function, such as the amygdala, insula, parabrachial nucleus, and solitary tract nucleus, as well as the peripheral distribution on the cardiac plexus^52,53^. Downregulated activity should lead to a lower sympathetic predominance, as observed in our population, with women exhibiting lower variation in the SDNN, shorter mean RR intervals, and greater power in the HF band (Table 2). These results also align with previous findings of decreased adrenergic sensitivity, increased vagal outflow, and a slower response to sympathetic activity in females^54,55^.

On the other hand, in addition to sex-dependent autonomic regulation, there is further sexual dimorphism in the pattern of expression of circadian-related genes in the heart. Women exhibit an oscillatory expression of focal adhesion genes such as *COL1A2.* Men exhibit oscillatory expression of genes related to the tricarboxylic acid cycle, fatty acid oxidation, glycogenolysis, and increased oscillation of mRNAs^47^. These genetic characteristics determine structural and metabolic patterns, contributing to sex-specific phenotypes and cardiac adaptations that potentially lead to differences in susceptibility to cardiovascular diseases and responses to treatment^56,57^. Thus, these differential oscillations may explain the later onset of the stress response and the earlier parasympathetic activity observed in males (Table 2).

### Limitations

The retrospective nature of this protocol may introduce information bias. To address this, we implemented strict inclusion criteria, conducted stratified analyses, and incorporated all available variables from the database.

Real-life recordings often face challenges in both acquisition and processing. To mitigate these issues, we applied combined strategies, including manual artifact removal and visual RR interval detection, using Kubios HRV Scientific’s built-in noise detection and threshold-based beat correction algorithms, and carefully selected analysis windows. Nevertheless, as we relied on a previously published database, it was not possible to control participants’ physical activity levels or daily routines during data collection.

Our assessment of sex-specific effects on circadian HRV dynamics is limited by the need for more data regarding reproductive phases, which could influence autonomic patterns. Future studies should include this information to explore detailed fluctuations throughout the reproductive cycle and enhance our understanding of neurohumoral differences between sexes.

## Concluding remarks

Together, our results support the hypothesis of interconnectivity between aging, sex and autonomic cardiac regulation during the circadian cycle. We argue that this connection has important implications for clinical practice. For example, the decline in electrical heart complexity with aging underscores the vulnerability of the senescent heart to arrhythmias and other cardiovascular dysfunctions^21,45,58^, which may arise from diminished adaptability to physiological demands^19,59,60^. Moreover, the delayed sympathetic activation and increased parasympathetic predominance observed in women may confer a protective effect against heart remodeling, arrhythmias, and other stress-induced cardiac events; however, it could also predispose them to bradycardia and hypotensive episodes. These sex-specific autonomic patterns underscore the need for tailored clinical interventions to address the unique vulnerabilities in cardiac aging and optimize treatment outcomes between sexes.

The successful implementation of our method to assess how intrinsic and extrinsic factors influence heart function is also relevant. We demonstrate that it is possible to detect oscillatory activity and circadian patterns on the basis of real-life ECG signals. Previous studies have focused on analyzing linear heart rate variability parameters and identifying general trends during the 24-hour cycle^55,61^. In contrast, our approach allows for an exhaustive circadian assessment of linear and nonlinear variables, leveraging continuous ECG data to capture subtle oscillatory dynamics influenced by sex and age. Using circadian parameters of HRV metrics, we successfully compared and analyzed the effects of physiological variables on autonomic cardiac activity throughout the 24-hour cycle. The insights resulting from our method should motivate further research on the circadian patterns in patients with cardiovascular diseases. This study further highlights the relevance of individualized approaches to cardiovascular health and the need for age-specific and sex-specific therapeutic strategies to maintain or restore autonomic balance and support healthy circadian rhythms in cardiac function.

## Supplementary Information

Below is the link to the electronic supplementary material.

Supplementary Material 1

## Data Availability

The data that support the findings of this study are available upon reasonable request from the corresponding author.
