# Supplementary material for: Age and sex affect circadian patterns of cardiac autonomic function
Source: Sci Rep. 2025 Sep 29;15:33677. doi: 10.1038/s41598-025-18525-6 (PMC12480707; doi:10.1038/s41598-025-18525-6)
Supplement: Supplementary file 1 — Supplementary Material 1 [file 41598_2025_18525_MOESM1_ESM.docx]

**Supplementary material**

| **Analysis** | **Variable** | **ANOVA** |
| --- | --- | --- |
|  | SDNN | F_23,86_= 2.61, p<0.001 |
|  | RMSSD | F_23,86_= 3.73, p<0.001 |
|  | pNN50 | F_23,86_= 4.95, p<0.001 |
| Frequency domain(4) | HF | F_23,86_= 4.88, p<0.001 |
|  | LF | F_23,86_= 4.84, p<0.001 |
|  | VLF | F_23,86_= 1.65, p=0.02 |
|  | LF/HF | F_23,86_= 19.07, p<0.001 |
| Non-linear(9) | SD1 | F_23,86_= 3.73, p<0.001 |
|  | SD2 | F_23,86_= 1.97, p=0.03 |
|  | SD2/SD1 | F_23,86_= 3.68, p<0.001 |
|  | SamPEN | F_23,86_= 3.11, p<0.001 |
|  | AmpEN | F_23,86_= 1.68, p=0.02 |
|  | Alf1 | F_23,86_= 3.79, p<0.001 |
|  | Alf2 | F_23,86_= 3.75, p<0.001 |
|  | CorrDim | F_23,86_= 1.76, p=0.01 |
|  | REC | F_23,86_= 1.10, p=0.32 |
|  | DET | F_23,86_= 2.12, p=0.001 |
|  | ShannE | F_23,50_= 1.21, p=0.223 |
| Autonomic indexes(3) | SNS | F_23,86_= 7.14, p<0.0001 |
|  | PNS | F_23,86_= 11.72, p<0.0001 |

**Table 1. Repeated measures ANOVA- rhythmicity test**


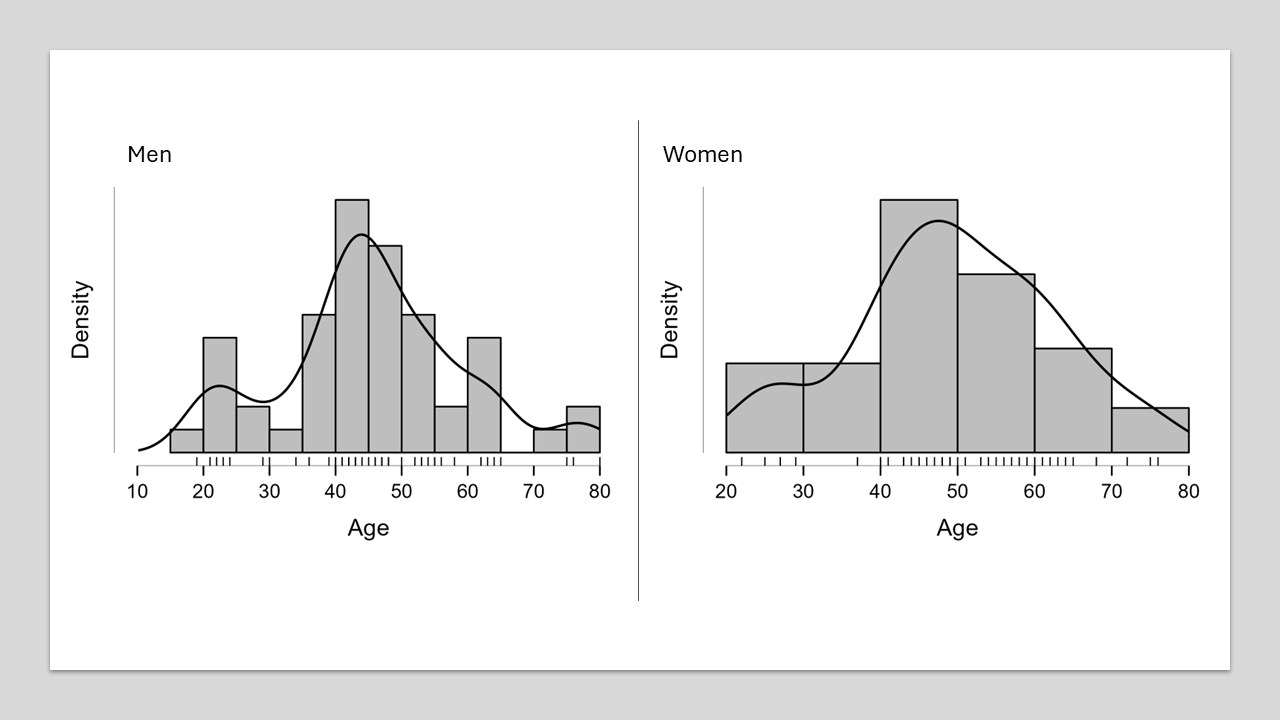


**Figure 1. Histograms of age distribution by sex**


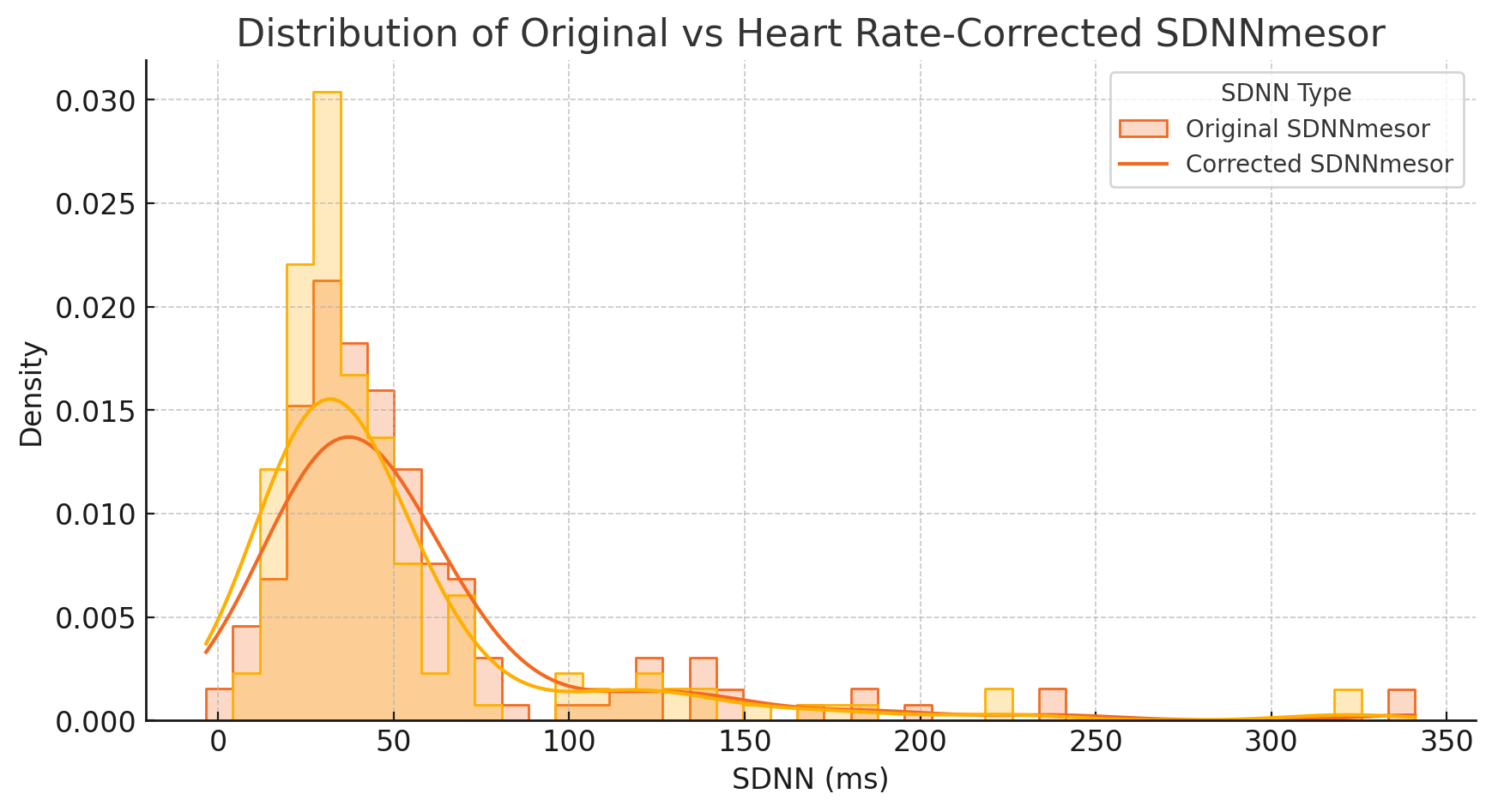


**Figure 2. Distribution of original vs HR-corrected SDNN MESOR**

**
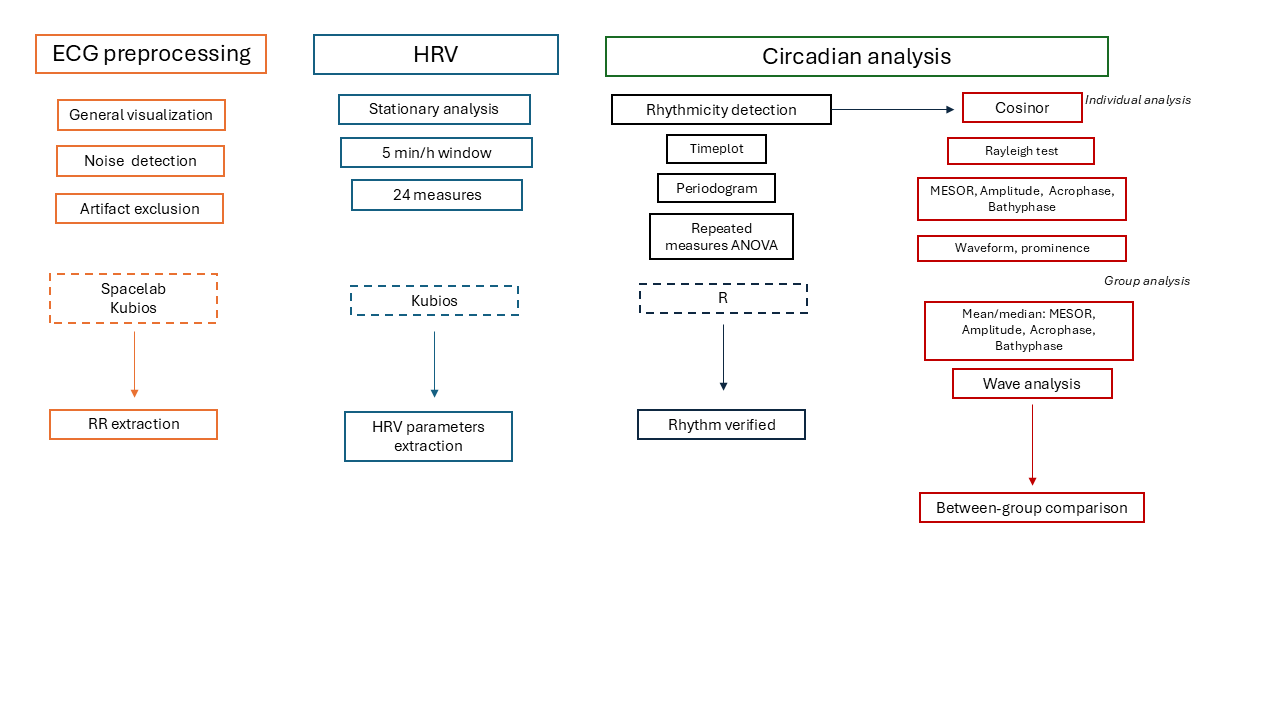
**

**Figure 3. Analysis procedures**
